# ABT-263 Enhances Sensitivity to Metformin and 2-Deoxyglucose in Pediatric Glioma by Promoting Apoptotic Cell Death

**DOI:** 10.1371/journal.pone.0064051

**Published:** 2013-05-17

**Authors:** Jane Levesley, Lynette Steele, Claire Taylor, Priyank Sinha, Sean E. Lawler

**Affiliations:** 1 Translational Neuro-Oncology Group, Leeds Institute of Molecular Medicine, University of Leeds, St James’s University Hospital, Leeds, United Kingdom; 2 Genomics Facility, Leeds Institute of Molecular Medicine, University of Leeds, St James’s University Hospital, Leeds, United Kingdom; University of Colorado, School of Medicine, United States of America

## Abstract

Pediatric high grade glioma is refractory to conventional multimodal treatment, highlighting a need to develop novel efficacious therapies. We investigated tumor metabolism as a potential therapeutic target in a panel of diverse pediatric glioma cell lines (SF188, KNS42, UW479 and RES186) using metformin and 2-deoxyglucose. As a single agent, metformin had little effect on cell viability overall. SF188 cells were highly sensitive to 2-deoxyglucose however, combination of metformin with 2-deoxyglucose significantly reduced cell proliferation compared to either drug alone in all cell lines tested. In addition, the combination of the two agents was associated with a rapid decrease in cellular ATP and subsequent AMPK activation. However, increased cell death was only observed in select cell lines after prolonged exposure to the drug combination and was caspase independent. Anti-apoptotic BCL-2 family proteins have been indicated as mediators of resistance against metabolic stress. Therefore we sought to determine whether pharmacological inhibition of BCL-2/BCL-xL with ABT-263 could potentiate apoptosis in response to these agents. We found that ABT-263 increased sensitivity to 2-deoxyglucose and promoted rapid and extensive cell death in response to the combination of 2-deoxyglucose and metformin. Furthermore, cell death was inhibited by the pan-caspase inhibitor, z-VAD-FMK suggesting that ABT-263 potentiated caspase-dependent cell death in response to 2-deoxyglucose or its combination with metformin. Overall, these data provide support for the concept that targeting metabolic and anti-apoptotic pathways may be an effective therapeutic strategy in pediatric glioma.

## Introduction

Pediatric high grade glioma comprises a heterogeneous group of brain tumors which are refractory to conventional multimodal therapy [1], [2], [3], [4]. Although very young children diagnosed with high grade glioma have been reported to have an improved outcome in comparison to older patients [4], the overall clinical outlook remains poor with 2-year survival rates ranging from 10–30% [2], [3]. Furthermore, survivors are often seriously compromised due to the lasting effects of radiation and surgery, highlighting an urgent need to develop more effective and less toxic therapies.

The therapeutic targeting of cancer metabolism has become a major area of investigation and is largely based on the principle that cancer cells display increased glucose uptake and production of lactate, even in the presence of adequate oxygen. This is known as the Warburg effect and suggests a dependency on aerobic glycolysis in rapidly growing tumors [5], [6], [7]. However, recent studies in intact brain tumors and human orthotopic mouse models of glioblastoma have demonstrated that their metabolism *in vivo* involves extensive mitochondrial oxidation of glucose [8], [9]. These findings indicate both glycolysis and mitochondrial glucose oxidation are necessary to support the rapid and aggressive growth observed in high grade glioma [10]. Furthermore, mitochondrial metabolism has been linked to drug resistance in glioblastoma, as DNA damaging agents have been shown to induce a cytoprotective ATP surge via oxidative phosphorylation [11]. These data indicate that therapeutic strategies directed against the metabolism of these tumors may need to target both glycolysis and mitochondrial oxidative phosphorylation in order to be effective.

Metformin (1,1 dimethylbiguanide hydrochloride) is a widely used anti-diabetic agent that has been shown to possess anti-cancer activity in a variety of tumor models [12], [13], [14], [15], [16], [17]. Whilst some studies have demonstrated that metformin may have anti-glioma action and enhance the efficacy of temozolomide treatment [18], [19] the effects of metformin on pediatric glioma cells have not been investigated previously. 2-deoxyglucose (2DG) is a glucose analog that is readily taken up by glucose transporters and acts as a competitive inhibitor of glycolysis [20]. The combination of metformin with 2DG has been shown to impair metabolism and induce cell death in multiple tumor types [21], [22], [23]. 2DG and metformin have been shown to decrease cellular ATP and induce an apoptotic form of cell death or a sustained autophagic response depending on the cellular context [21], [22]. These effects have been attributed to a simultaneous block of glycolysis (with 2DG) and oxidative phosphorylation due to the ability of metformin to partially suppress the activity of complex I of the mitochondrial respiratory chain [21]. Based on these preclinical studies it has been proposed that the combination of 2DG and metformin may be an effective treatment for some cancer types, however, it has not yet been tested in brain tumors.

In this study, we initially investigated the effects of metformin and 2DG on a diverse panel of well characterised pediatric glioma cell lines. Our results demonstrate that in the majority of cell lines studied, the combination of metformin and 2DG decreases pediatric glioma cell proliferation. However, increased cell death was only observed in specific cell lines after lengthy periods of exposure to the drug combination and was caspase independent. We found that sensitivity to the combination of 2DG and metformin was greatly enhanced by the selective BCL-2/BCL-xL inhibitor ABT-263, which promoted caspase-dependent cell death in all cell lines. We conclude that metabolic therapy in pediatric high grade glioma is greatly enhanced by impairment of BCL-2/BCL-xL function, and that future therapies should be developed based on this approach.

## Materials and Methods

### Cell Culture

Previously characterized pediatric glioma cell lines SF188, KNS42, UW479 and RES186 were donated by Dr Chris Jones (Institute of Cancer Research, London, United Kingdom) [24]. All cell lines were cultured in DMEM containing 10% (v/v) fetal bovine serum (FBS) at 37°C in 5% CO_2_.

### Chemicals

All chemicals were purchased from Sigma-Aldrich unless otherwise stated. 2DG and metformin were prepared as 500 mM solutions in sterile distilled H_2_O. Stock solutions of ABT-263 (10 mM; Selleckchem) and z-VAD-FMK (25 mM; BD) were prepared in DMSO.

### Cell Viability and Proliferation Assays

Cells were seeded in triplicate at a density of 5×10^3^ cells per well in 96-well plates and left to attach overnight prior to treatment. Cell viability was determined by cleavage of the soluble WST-1 substrate (Roche) followed by spectrophotometric measurement of the product at 450 nm. The CyQuant cell proliferation kit (Invitrogen) was used to determine cell density after drug treatment, according to the manufacturers’ instructions. Fluorescence was measured using a Mithras LB 940 multimode plate reader (Berthold Technologies) using standard filter sets for FITC (Ex = 485 nm/Em = 535 nm).

### Measurement of ATP

Total cellular ATP was determined using the CellTitre-Glo luminescence assay (Promega) according to the manufacturers’ instructions. 5×10^3^ cells per well were plated in triplicate in opaque, white-walled 96-well plates (Corning). Cells were treated as appropriate, lysed using a volume of CellTitre-Glo reagent equal to the total culture volume and luminescence measured using a Mithras LB 940 multimode plate reader (Berthold Technologies).

### Flow Cytometry Analysis of Cell Death

Membrane integrity was determined by flow cytometric quantification of propidium iodide uptake. Cells were seeded in triplicate at a density of 5×10^4^ cells per well in 24-well plates. After treatment, cells were harvested by trypsinization and washed with ice-cold PBS containing sodium azide (0.1% v/v). Cell pellets were re-suspended in PBS/sodium azide solution containing propidium iodide (0.83 µg/ml) and analysed by flow cytometry. Apoptosis was measured using an Annexin-V-FITC/PI apoptosis detection kit (BD). Briefly, 1×10^5^ cells were plated in 12 well plates and treated as appropriate. Cells were harvested using accutase detachment solution (Sigma) and Annexin-V-FITC/PI labelling was performed according to the manufacturers’ instructions. Quantification of Annexin-V/propidium iodide incorporation was performed using a FACScalibur flow cytometer. Data were analysed using FACSDiva software 6.0 (BD) and winMDI2.8.

### Quantification of Caspase 3/7 Activity

Caspase activity was assayed by cleavage of a luminogenic caspase 3/7 substrate containing the sequence DEVD, using the caspase 3/7-Glo kit (Promega) according to manufacturers’ instructions. 5×10^3^ cells per well were plated in triplicate in opaque, white-walled 96-well plates (Corning). Cells were lysed using a volume of Caspase3/7-Glo reagent equal to the total culture volume. Luminescence was measured using a Mithras LB 940 multimode plate reader.

### Western Blotting

Cells were seeded in 100 mm dishes (Falcon) and allowed to adhere overnight prior to treatment. Whole cell lysates were prepared and separated by SDS-PAGE, electroblotted to nitrocellulose membrane (GE Healthcare Life Sciences) and probed with antibodies against PARP, caspase-3, BCL-xL, BCL-2, BCL-w, AMPKα and phospho-AMPKα (Thr172) (Cell Signalling Technologies) and MCL-1 (Santa Cruz Biotechnology). Antibody binding was detected using the Odyssey system (LI-COR Biosciences). Equal lane loading was confirmed using a monoclonal antibody against β-actin (Sigma-Aldrich).

### Immunocytochemistry

Conformational activation of BAX was detected by immunocytochemistry using the anti-BAX 6A7 antibody (Enzo Lifesciences). Cells were grown on chamberslides (Labtek), treated as desired and fixed with 4% (w/v) paraformaldehyde for 20 minutes at room temperature. A blocking step was carried out using 20% (v/v) normal goat serum in PBS for 1 h at room temperature. Cells were incubated with anti-BAX 6A7 (1∶200) in PBS containing 2% (v/v) normal goat serum and 0.1% (v/v) Triton-X100, overnight at 4°C. Anti-BAX was detected using an AlexaFluor 555 anti-mouse antibody (Invitrogen), prepared in blocking buffer (5% (v/v) normal goat serum/PBS) at a dilution of 1∶300 for 1 h at room temperature. Cells were mounted in Vectashield containing DAPI (Vector) to visualise nuclei.

### Statistical Analysis

SPSSv16 was used to analyse quantitative data from independent experiments. Statistical significance between multiple groups was determined by Kruskal-Wallis ANOVA tests which were followed up with Bonferroni-corrected Mann-Whitney U tests for paired comparisons.

## Results

### Metformin and 2DG Combination Impairs the Growth of Pediatric Glioma Cell Lines

**Figure 1:**
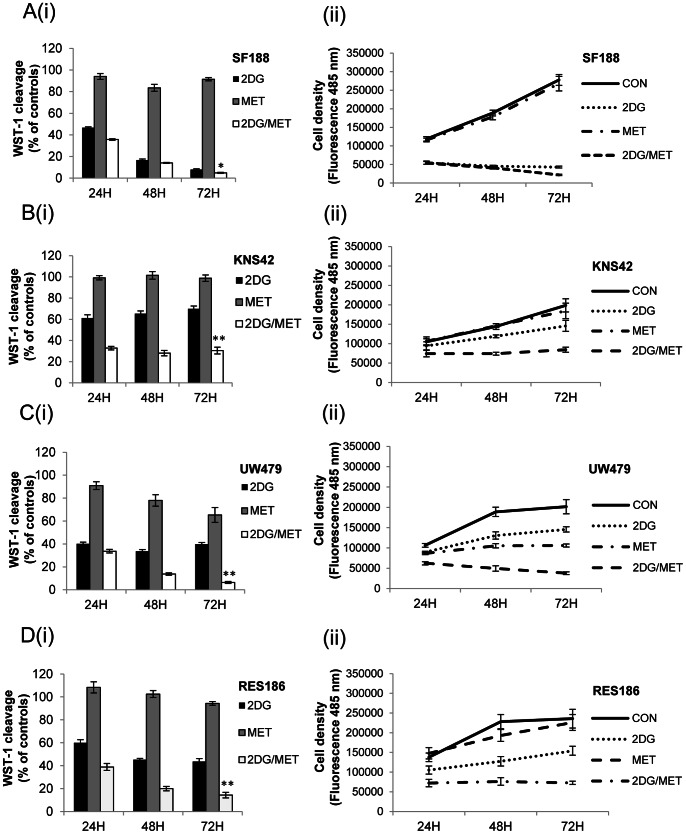
Metformin inhibits the growth of pediatric glioma cell lines in response to 2DG. Pediatric glioma cells were cultured in 25 mM glucose in the presence of 2DG (10 mM), metformin (8 mM) either alone or in combination. Cell viability was assessed by WST-1 cleavage after 24, 48 and 72 hours (A(i)–D(i)), whilst effects on cell proliferation were measured by fluorescent quantification of cell density (A(ii)–D(ii)). For WST-1 assays results are expressed as mean percentages relative to mock treated cells. Error bars represent the standard error of the mean from three independent experiments, repeated in triplicate. Statistical significance was determined using Kruskal-Wallis ANOVA followed by Bonferroni corrected Mann-Whitney U tests: *P≤0.05, **P≤0.01, 2DG and metformin treated cells vs. single agents.

In order to examine the potential of cellular metabolism as a therapeutic target in pediatric glioma, we investigated the effect of the drugs metformin and 2DG on a diverse panel of previously characterized cell lines (SF188 and KNS42; grade IV glioblastoma multiforme, UW479; grade III anaplastic astrocytoma and RES186; grade I pilocytic astrocytoma) [24]. We found that all of these cell lines possessed homozygous *TP53* mutations, and that KNS42 carried the heterozygous *H3F3A* mutation (G34V; Figure S1) recently discovered in pediatric glioblastoma [25], [26]. First, we examined the effects of metformin and 2DG on cell viability and cell proliferation alone and then in combination (Figure 1A(i)–D(i)). Treatment with 8 mM metformin alone had little effect on cell viability as determined by WST-1 cleavage under standard growth conditions whereas this was somewhat reduced following incubation with 10 mM 2DG alone. SF188 cells were highly sensitive to 2DG with only 7.9±0.7% of viable cells remaining after 72 hours of treatment (Figure 1A(i)). In the remaining cell lines, WST-1 cleavage was moderately reduced by 2DG treatment (69±2.5%–43.6±2.5%) compared with controls (Figure 1B(i)–1D(i)). However, combination of metformin with 2DG led to a significant further reduction in WST-1 cleavage in each cell line. The enhancing effect of the drug combination at 72 hours was greatest in UW479 (6.4±0.8%, Figure 1C(i)), followed by RES186 (14.3±2.4%, Figure 1D(i)) and KNS42 cells (30.5±3.2%, Figure 1B(i)). Even in SF188 cells, the combination showed a small but significant difference compared with 2DG alone (4.9±0.4% versus 8.0±0.8%, *P = 0.006). In order to directly assess the effects of 2DG and metformin upon cell proliferation we measured cell density after 24, 48 and 72 hours of treatment (Figures 1A(ii)–D(ii)). In response to the single treatments, 2DG had the most profound effect on SF188 cells, whilst growth was most impaired in UW479 cells following treatment with metformin alone. However, in all cell lines combination of metformin with 2DG resulted in a significant reduction in cell growth over the time course (**P≤0.002). Overall, these data show that the combination of 2DG and metformin inhibits tumor cell proliferation significantly better than either drug alone in all the pediatric glioma cell lines tested.

### Combined Metformin and 2DG Treatment Results in ATP Depletion and Phosphorylation of AMPK

**Figure 2:**
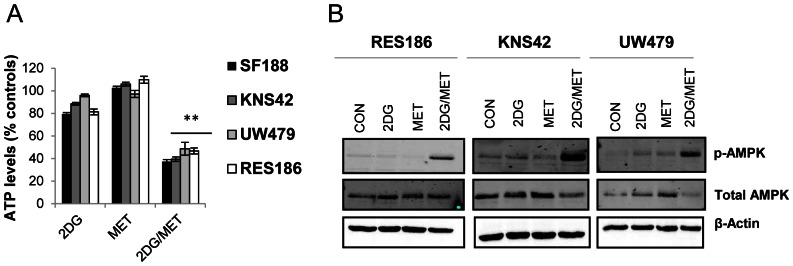
Metformin and 2DG treatment results in a decrease in ATP levels and phosphorylation of AMPK. (A) ATP levels were measured after 8 hours of treatment with 2DG (10 mM), metformin (8 mM) either alone or in combination. Data are presented relative to vehicle treated cells (mean ± SEM; n = 3 experiments, repeated in triplicate; Kruskal-Wallis ANOVA followed by Bonferroni corrected Mann-Whitney U tests: **P≤0.01, 2DG and metformin treated cells vs. single agents). (B) Lysates from cells treated with 2DG (10 mM), metformin (8 mM) or a combination, for 96 hours were immunoblotted using antibodies against p-AMPKα and total AMPKα. Representative blots from three independent experiments are shown.

To determine the effects of 2DG and metformin on cellular energetics more directly, we assayed ATP levels after a brief period of treatment of 8 hours (Figure 2A). Metformin treatment had little impact on ATP levels in the cell line panel even after sustained treatment for 96 hours (data not shown). Impairment of glycolysis with 2DG was sufficient to reduce ATP levels to 88.4±1.3%–79.4±1.2% within 8 hours of treatment, with SF188 cells displaying the largest decrease in ATP content (Figure 2A). However, the dual combination was much more effective, causing a significant rapid reduction in cellular ATP content to 46.7±2.6%–37.0±2.4% within 8 hours of treatment in all cell lines (combination versus single treatments: **P<0.001). We obtained similar results when we investigated the effects of 2DG and metformin treatment on AMPK phosphorylation which is an indicator of cellular metabolic stress (Figure 2B). Continuous exposure to 2DG or metformin alone failed to induce activation of AMPK, suggesting that the cellular AMP/ATP ratio was not critically affected. However, we observed robust phosphorylation of AMPK at Thr-172 in cells treated with both 2DG and metformin, supporting our observation that the combination caused a decline in total cellular ATP levels. Overall, these data suggest that ATP production is only substantially impaired following treatment with both metformin and 2DG in paediatric glioma cells.

### Combined Metformin and 2DG Treatment Leads to Cell Death or Growth Arrest

**Figure 3:**
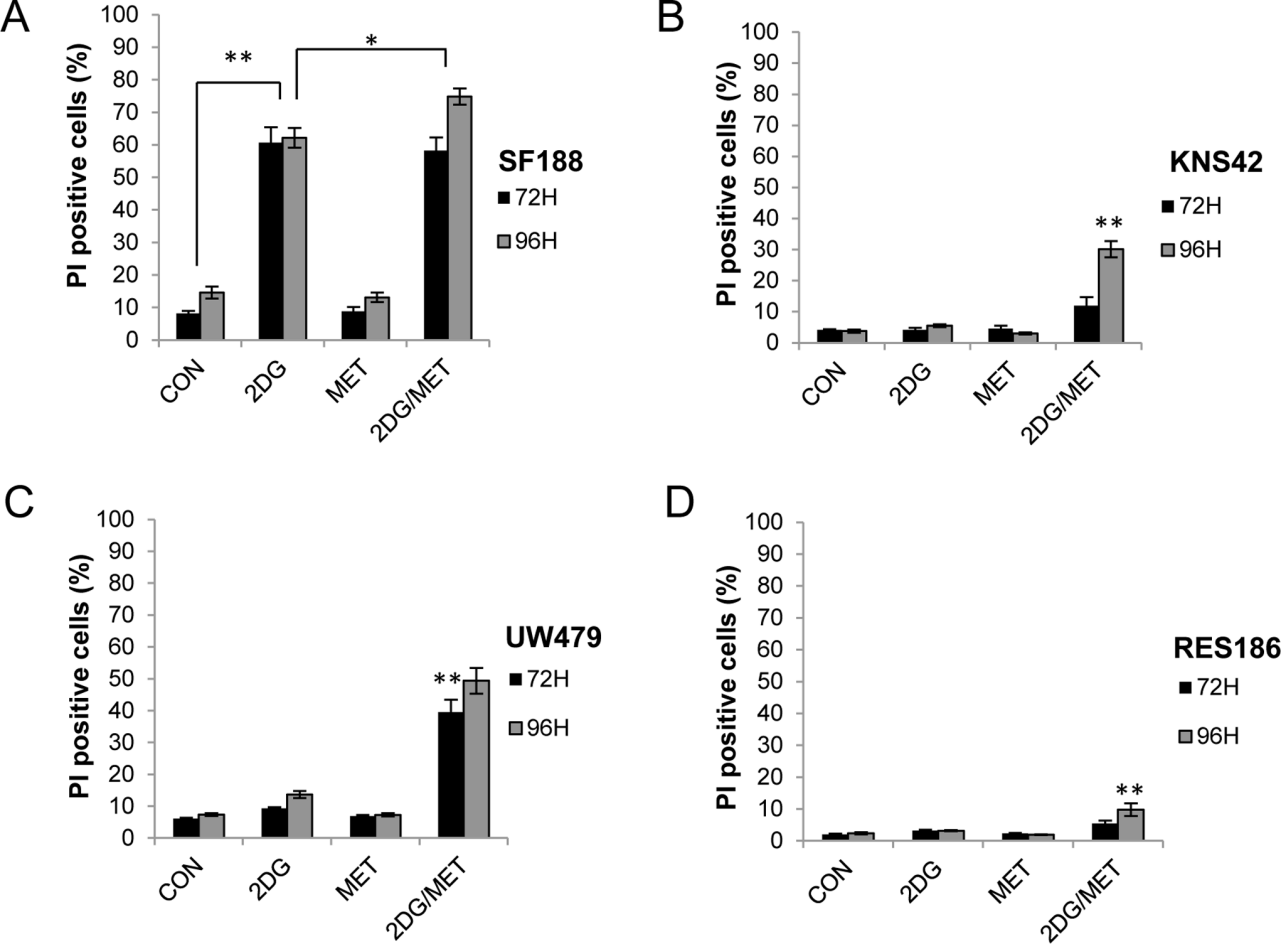
Metformin and 2DG treatment results in cell death or inhibition of proliferation. (A–D) Viability of glioma cells treated with 2DG (10 mM), metformin (8 mM) either alone or in combination was determined by propidium iodide exclusion after 72 and 96 hours (mean ± SEM; n = 3 experiments, repeated in triplicate; Kruskal-Wallis ANOVA followed by Bonferroni corrected Mann-Whitney U tests: *P≤0.05, **P≤0.01, control vs. 2DG treated cells and 2DG treated cells vs. 2DG/metformin combination therapy).

In order to determine the effects of metformin and 2DG treatment on cell death, we used flow cytometry to assess membrane integrity via propidium iodide uptake. Metformin alone had no effect upon cell death compared to vehicle controls whilst 2DG alone only caused significant levels of cell death in the highly sensitive SF188 cell line (Figure 3A). Only the combination of 2DG and metformin significantly increased cell death in UW479 cells after 72 hours (39.4±4.0%, **P<0.001; Figure 3C). In the KNS42 and RES186 cell lines, the proportion of cells exhibiting a loss of membrane integrity after 72 hours was not significantly increased, confirming that the effects of 2DG and metformin observed previously (Figure 1) were due to decreased cellular proliferation in the first instance. However, in KNS42 cells, increased cell death was observed after 96 hours, with 30±2.6% of cells displaying a loss of membrane integrity following treatment with 2DG and metformin (Figure 3B). By contrast, in RES186 cells, the combination of 2DG and metformin even at 96 hours did not induce significant levels of cell death, confirming that the combination of metformin and 2DG exerted an anti-proliferative effect in this cell line (Figure 3D). Examination of the DNA profiles of RES186 cells treated with either agent alone or in combination revealed that 2DG and metformin led to an S/G2-M phase cell cycle accumulation as opposed to cell death (data not shown).

### Cell Death Induced by the Combination of Metformin and 2DG Treatment is Caspase Independent

**Figure 4:**
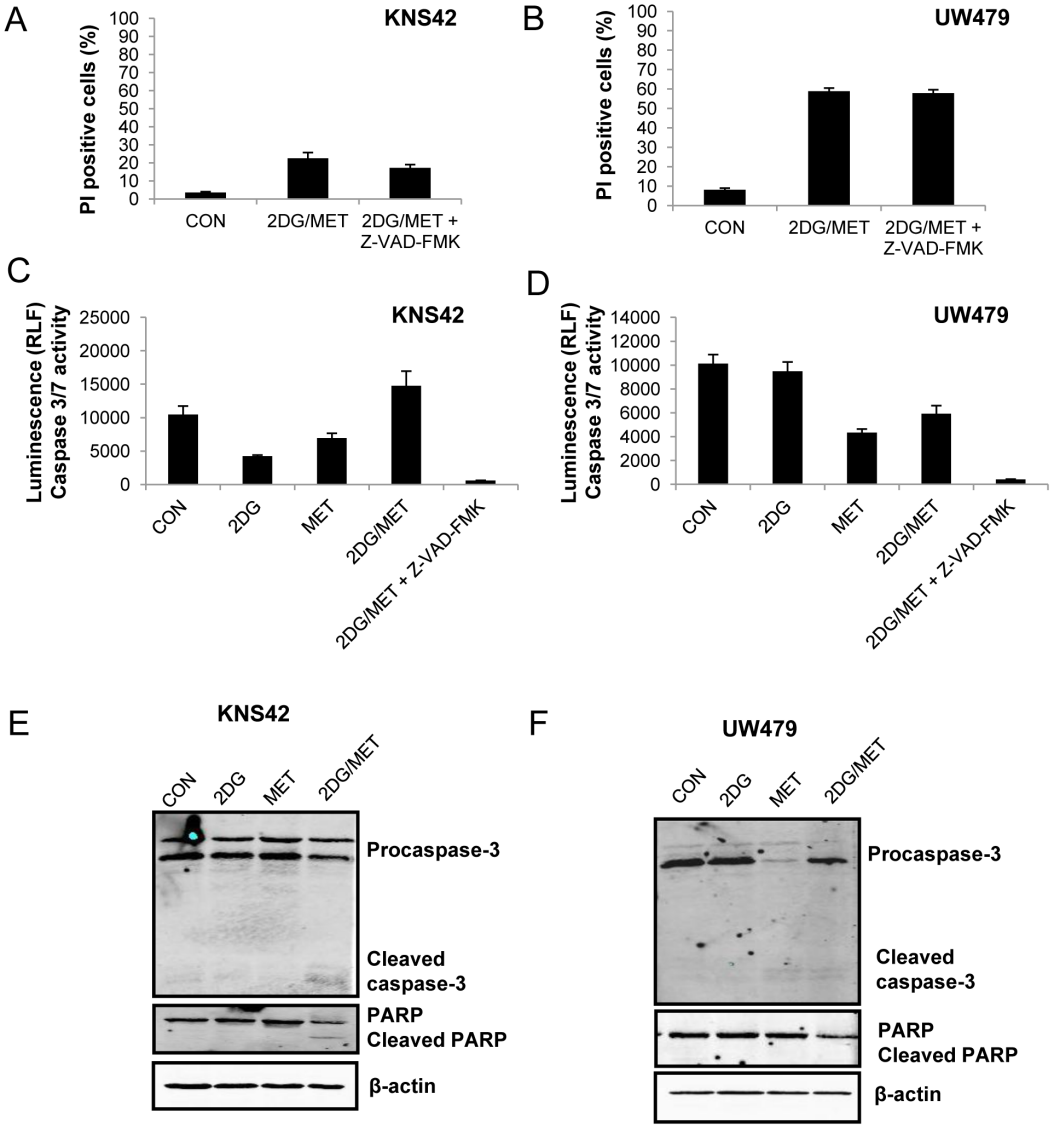
Cell death induced by 2DG and metformin is caspase independent. (A–B) KNS42 and UW479 cells were incubated with the pan-caspase inhibitor, z-VAD-FMK (50 µM) for 1 hour prior to addition of 2DG (10 mM), metformin (8 mM) or both agents and cultured for 96 hours. Cell viability was determined by propidium iodide exclusion (mean ± SEM; n = 3 experiments, repeated in duplicate). (C–D) Caspase 3/7 activity was quantified by measuring cleavage of a luminogenic peptide which is a substrate for caspase 3/7. UW479 cells were treated for 72 hours with 2DG and metformin either alone or in combination. Cells were pre-treated with z-VAD-FMK (50 µM) for 1 hour prior to the addition of 2DG and metformin for 72 hours.(C) KNS42 cells were treated similarly and caspase 3/7 activity was assayed after 96 hours of exposure to 2DG, metformin or both agents. Cells were pre-treated with z-VAD-FMK (50 µM) for 1 hour prior to the addition of 2DG and metformin for 96 hours. (E–F) Lysates of treated cells were immunoblotted with anti-caspase 3 and PARP. Equal loading was confirmed using a β-actin antibody. Representative blots from three independent experiments are shown.

KNS42 and UW479 cells exhibited moderate levels of cell death in response to 2DG and metformin treatment and were selected for further investigation into the mechanism of cell death. Blockade of caspase activity by z-VAD-FMK failed to protect cells from death induced by 2DG and metformin, suggesting that the mode of death promoted by these agents was caspase independent (Figure 4A and B). These findings were confirmed by a lack of caspase 3/7 activation (Figure 4C and D). In addition, Western blots indicated that there was an absence, or a very low level of caspase-3 or PARP cleavage in response to 2DG and metformin treatment (Figure 4E and 4F). In conclusion, the combination of metformin and 2-deoxyglucose induced either predominantly caspase-independent cell death or exerted an anti-proliferative effect in paediatric glioma cells.

### The Effects of 2DG and Metformin on Cell Death are Enhanced by ABT263

Given that 2DG and metformin only induced cell death after prolonged treatment in the majority of cell lines, and proceeded in the absence of caspase activation, we considered how sensitivity to these agents might be enhanced. Overexpression of anti-apoptotic BCL-2 proteins can confer apoptotic resistance in malignant glioma [27]. The BCL-2 family is also known to regulate cell death in response to metabolic stress [28], [29] and BH3-mimetics which target anti-apoptotic BCL-2 family members may increase sensitivity to therapies targeting cellular metabolism [30], [31], [32]. Therefore, we examined the role of the anti-apoptotic BCL-2 family members using the BAD-like mimetic, ABT-263.

**Figure 5:**
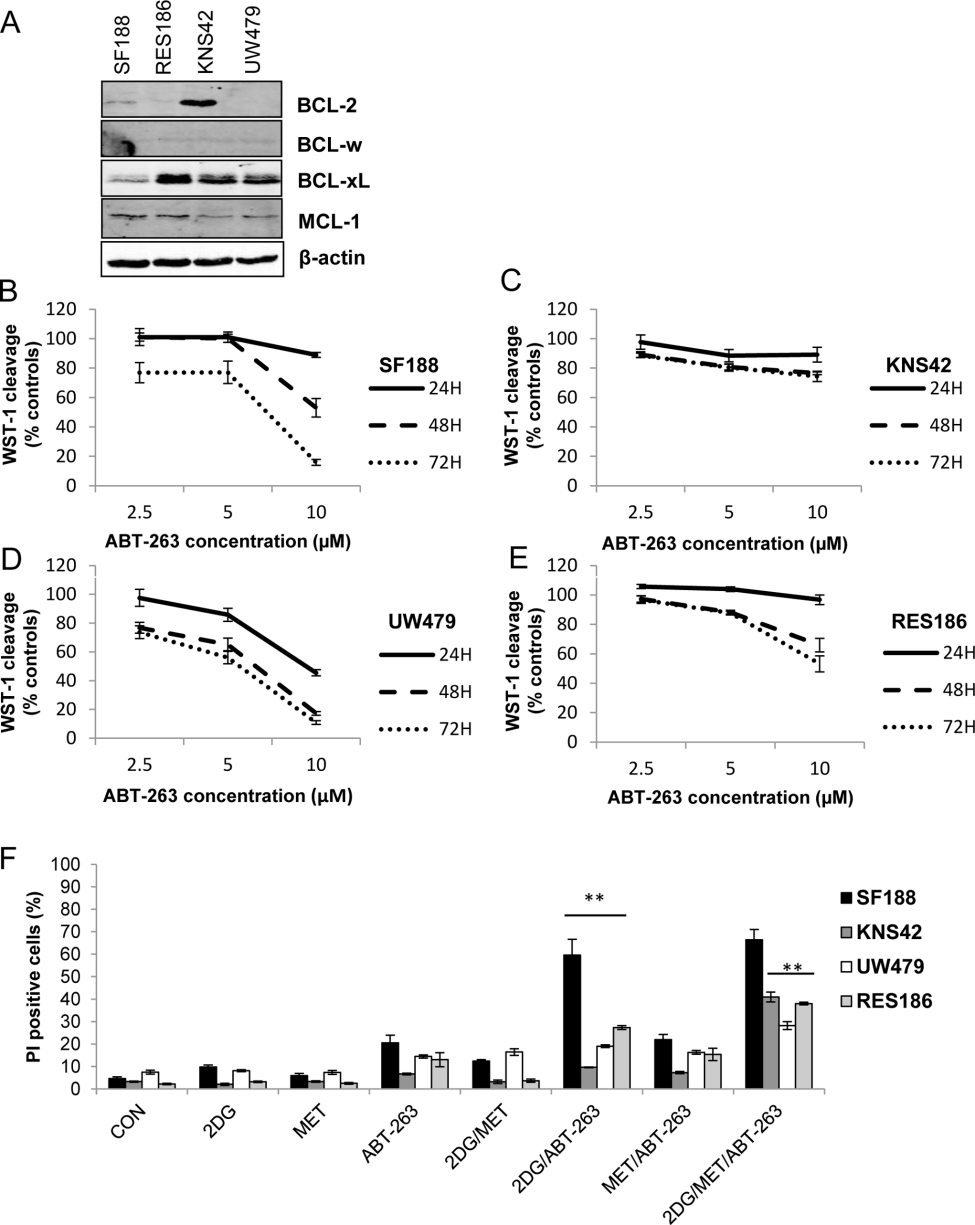
ABT-263 sensitizes pediatric glioma cells to 2DG and potentiates cell death in response to the combination of 2DG and metformin. (A) Lysates of pediatric glioma cells grown under standard culture conditions were immunoblotted with antibodies against BCL-2, BCL-xL, BCL-w and MCL-1. Representative blots from three independent experiments are shown. (B–E) Cells were treated with increasing concentrations of ABT-263 for 24–72 hours and viability was determined by measuring WST-1 cleavage (mean ± SEM; n = 3 experiments, repeated in triplicate). (F) Cells were treated with ABT-263 (10 µM), 2DG (10 mM), metformin (8 mM) alone and in combination. Membrane integrity was assessed by propidium iodide exclusion after 24 hours of treatment (mean ± SEM; n = 3 experiments, repeated in triplicate; Kruskal-Wallis ANOVA followed by Bonferroni corrected Mann-Whitney U tests: *P≤0.05, **P≤0.01, ABT-263 and 2DG treated cells vs. 2DG/ABT-263 treated cells and 2DG/ABT-263 combination vs. triple treatment).

Immunoblot analysis showed that BCL-2 and or BCL-xL were the major anti-apoptotic proteins present in the cell lines used in this study (Figure 5A). Exposure to 10 µM ABT-263 over 72 hours reduced viability in all cell lines as determined by WST-1 cleavage (Figure 5B–5E) with KNS42 cells, featuring overexpression of BCL-2 and BCL-xL, exhibiting the greatest resistance (Figure 5C). We then examined the ability of ABT-263 to potentiate cell death in response to 2DG, and metformin either alone or in combination (Figure 5F). Treatment with ABT-263 did not significantly increase cell death in combination with metformin under standard growth conditions after 24 hours. Interestingly, ABT-263 led to a small but significant increase in sensitivity to 2DG in the cell line panel, with a very pronounced effect in SF188 cells (59.6±7.0%). Finally, the combination of ABT-263 with both metformin and 2DG effectively promoted extensive cell death within 24 hours in all cell lines (KNS42, 40.9±2.2%; UW479, 28.2±1.2%; and RES186, 38±0.6%). This effect was not significant in SF188 cells which already displayed substantial sensitivity to 2DG and ABT-263 after 24 hours of treatment. These data suggest that the combination of metformin and ABT-263 increases the dependence of pediatric glioma cells on glycolysis for survival, since addition of 2DG promotes massive and rapid cell death.

### ABT-263 Promotes Caspase-dependent Cell Death in Combination with 2DG and Metformin

**Figure 6:**
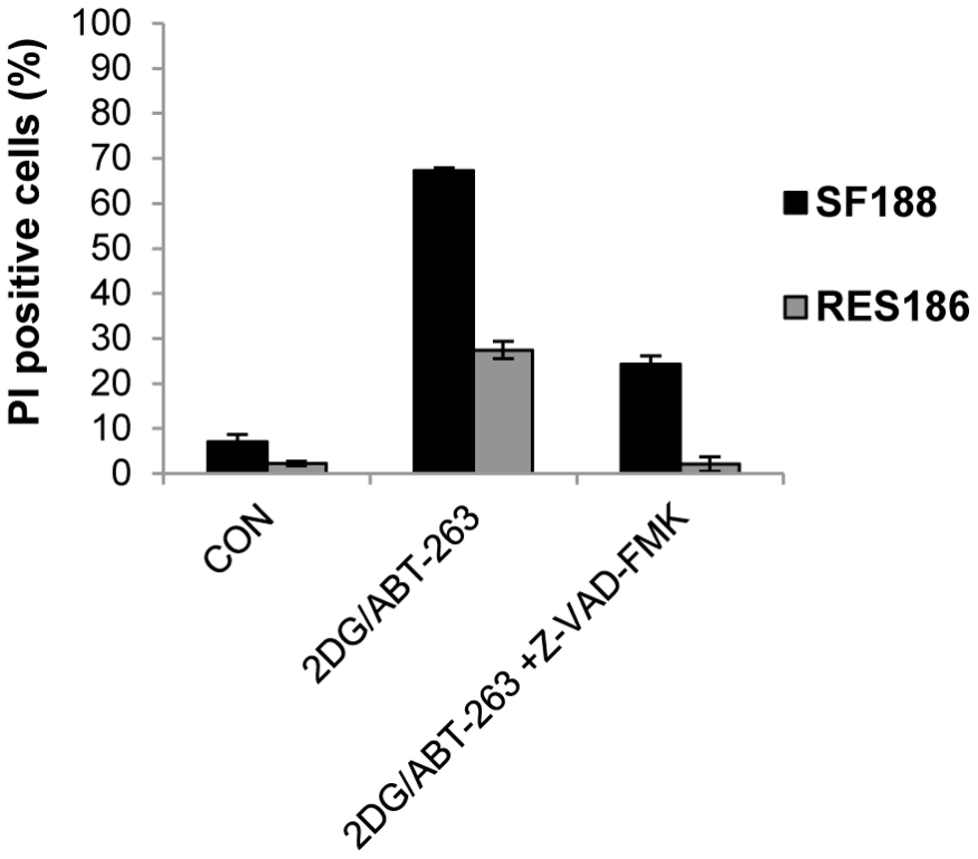
ABT-263 promotes caspase-dependent cell death in response to 2DG. (A) SF188 and RES186 cells were cultured in the presence of z-VAD-FMK (50 µM) for 1 hour prior to the addition of 2DG (10 mM) and ABT-263 (25 µM). Cells were treated for 24 hours and membrane integrity assessed following staining with propidium iodide (mean ± SEM; n = 3 experiments, repeated in triplicate).

**Figure 7:**
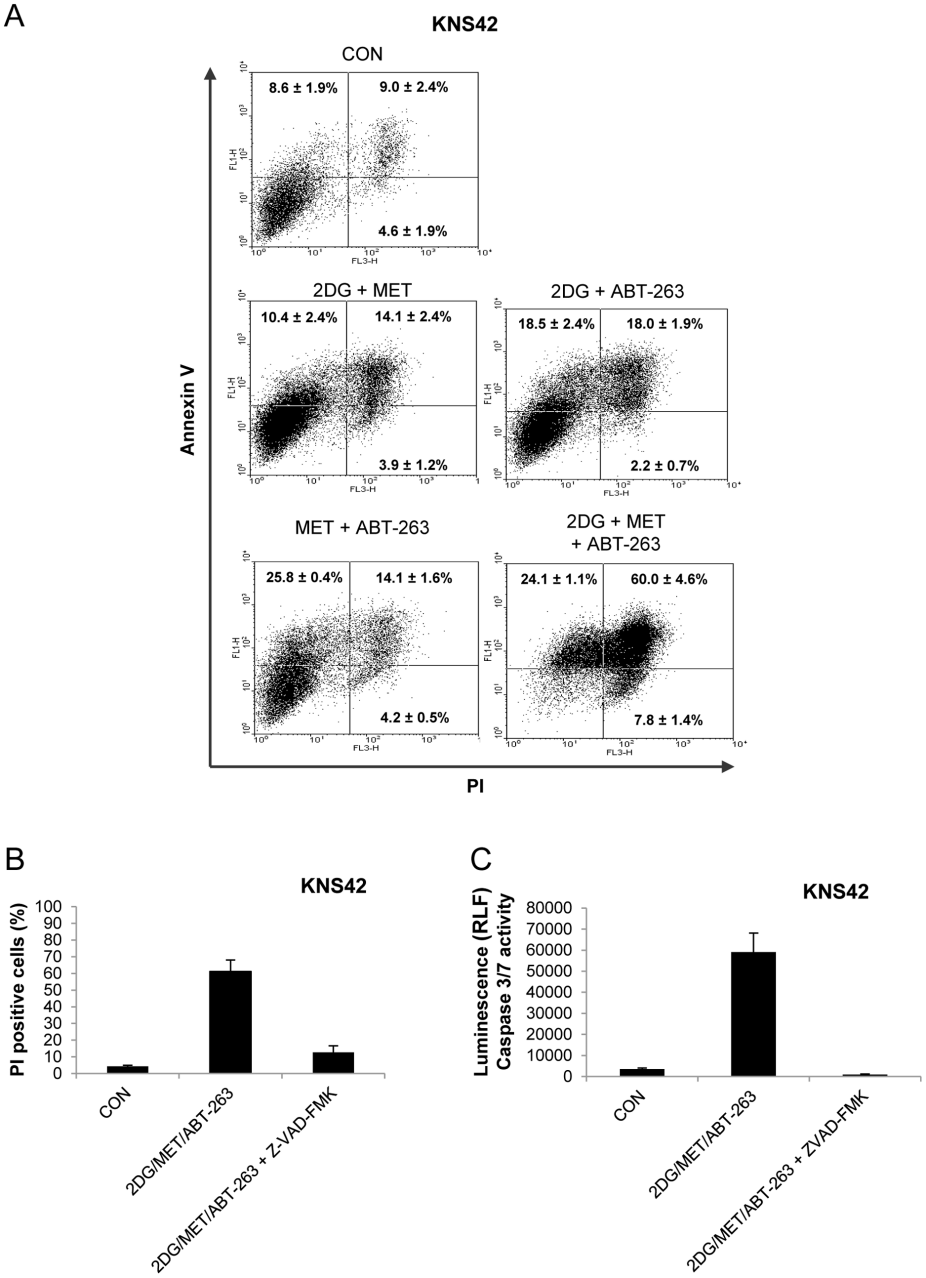
ABT-263 promotes caspase-dependent cell death in response to the combination of 2DG and metformin. (A) KNS42 cells were treated with a combination of 2DG (10 mM), metformin (8 mM) and ABT-263 (10 µM) for 16 hours and apoptosis was determined by Annexin-V/PI labelling. Representative dot plots are shown for each condition. Numerical data represent the mean ± SEM of three independent experiments. (B) KNS42 cells were incubated with z-VAD-FMK (50 µM) for 1 hour prior to the addition of 2DG (10 mM), metformin (8 mM) and ABT-263 (10 µM). Cells were treated for 24 hours and membrane integrity assessed following staining with propidium iodide. (C) Caspase 3/7 activity was quantified in KNS42 cells after 16 hours of treatment with 2DG (10 mM), metformin (8 mM) and ABT-263 (10 µM) in the presence or absence of z-VAD-FMK (50 µM). Cells were pre-treated with z-VAD-FMK for 1 hour prior to the addition of 2DG, metformin and ABT-263 (mean ± SEM; n = 3 experiments, repeated in triplicate).

**Figure 8:**
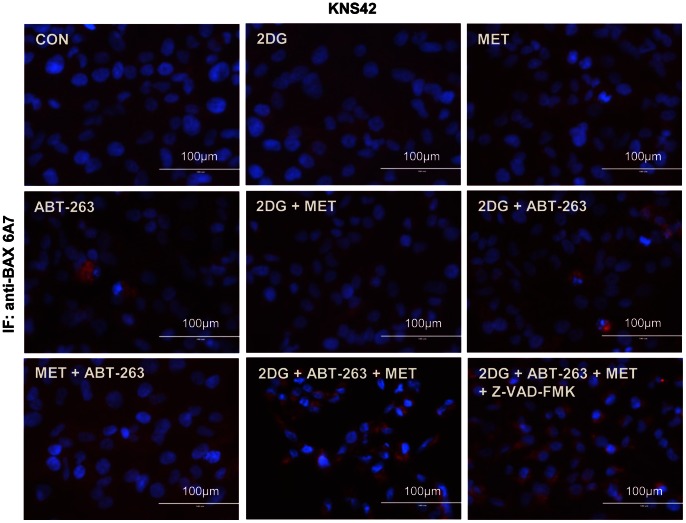
ABT-263 increases BAX activation during metformin and 2DG treatment. Activation of pro-apoptotic BAX in KNS42 cells was assessed via immunostaining using an antibody specific to active BAX after 8 hours of treatment with each agent or the various combinations. Representative images are shown from at least three independent experiments.

We used z-VAD-FMK to investigate the cell death pathways promoted by the ABT-263/2DG or ABT-263/2DG/metformin combinations (Figures 6 and 7). Inhibition of caspase activity significantly reduced cell death in SF188 and RES186 cells following 24 hours of exposure to 2DG and ABT-263 (Figure 6). In order to investigate the mechanism of cell death promoted by ABT-263 in response to the 2DG/metformin combination, we selected the KNS42 cell line for further study since it possesses the heterozygous *H3F3A* mutation found in pediatric glioblastoma. We confirmed by Annexin-V/PI staining that ABT-263 in combination with metformin and 2DG was much more effective at inducing apoptosis (84±4.3%) than the dual combination of either metformin/ABT-263 or 2DG/ABT-263 (Figure 7A). In addition, z-VAD-FMK significantly reduced cell death (Figure 7B) and completely abolished caspase 3/7 activity in response to the combination of all three agents in KNS42 cells (Figure 7C). Finally, we examined the effects of all agents and combinations upon BAX activation using immunocytochemistry and an antibody specific to the activated form of BAX (Figure 8). Active BAX immunostaining was generally faint and diffuse in cells treated with each agent or with the dual combinations. However, KNS42 cells treated with the triple combination of 2DG, metformin and ABT-263 exhibited strong, punctate staining of active BAX after 8 hours of treatment. Although we previously found that z-VAD-FMK abrogated cell death in response to all three agents it did not prevent activation of BAX. These data suggest that apoptosis induced by the combination of metformin, 2DG and ABT-263 proceeds via the mitochondria and requires downstream caspase activation. This data therefore supports a therapeutic paradigm in which metabolism and apoptosis are simultaneously targeted for the effective killing of pediatric glioma cells.

## Discussion

Tumor metabolism has become an important potential therapeutic target in cancer [6], [7], [29]. This is particularly relevant in aggressive brain tumors such as glioblastoma which have high metabolic demands. In this study we examined the concept of targeting tumour metabolism in a diverse panel of pediatric glioma cell lines using the glycolysis inhibitor 2DG, and metformin, a diabetes drug which has emerged as a promising anti-cancer agent.

Metformin has been shown to partially repress complex I of the respiratory chain and combination with 2DG attenuates tumour growth *in vitro* and *in vivo* [21], [22], [23]. We found that the response to 2DG varied in a cell line dependent manner, whilst metformin had little effect on cell viability overall. SF188 cells were highly sensitive to inhibition of glycolysis by 2DG which could be attributable to a variety of factors, including the *MYC* expression status of this cell line [33]. In agreement with other studies in prostate and colon carcinoma [21], [22], our data similarly indicates that glioma cells treated with metformin become increasingly dependent upon glycolysis because combination of metformin with 2DG resulted in a greater loss of cell viability than observed with 2DG alone. ATP levels were relatively unaffected by 2DG or metformin as single treatments, suggesting that cells were able to maintain energy levels using compensatory metabolic pathways. Under the conditions that we tested, only the combination of the two agents resulted in a substantial depletion of ATP and subsequent activation of AMPK. Overall, these results suggest that targeting individual metabolic pathways may be ineffective in glioma cells.

2DG and metformin have been reported to induce p53-dependent apoptosis in prostate cancer cell lines [21]. However, in our cell lines, which harbor homozygous *TP53* mutations, modest levels of cell death were only observed in UW479 and KNS42 cells after prolonged drug treatment of 72–96 hours. Furthermore, cell death induced by sustained 2DG and metformin treatment was not the result of apoptosis since it proceeded in the absence of caspase 3/7 cleavage and was not prevented by the pan-caspase inhibitor, z-VAD-FMK. In addition, whilst 2DG and metformin treatment attenuated the growth of all cell lines, it did not induce cell death in all cell lines, with RES186 cells exhibiting cell cycle arrest in the absence of cell death. The exception to this was the SF188 cell line, which was highly sensitive to 2DG treatment alone.

In order to investigate mechanisms of comparative resistance to the 2DG and metformin combination in the remaining cell lines, we investigated a role for the anti-apoptotic BCL-2 family proteins using the BH3-mimetic, ABT-263. Tumors over-expressing anti-apoptotic proteins such as BCL-xL may be endowed with an increased metabolic capacity to survive in nutrient deprived microenvironments by enhancing the efficiency of mitochondrial metabolism [34]. In addition, impairment of glycolysis and oxidative phosphorylation decreases ATP levels and sensitizes tumour cells to apoptotic stimuli [30]. Therefore, targeting these anti-apoptotic proteins may increase sensitivity to therapies targeting tumor metabolism. The closely related drug, ABT-737 has been shown to promote apoptosis in combination with 2DG in variety of cell lines [30], [31], [32]. We found that SF188 cells expressed endogenous BCL-2 and BCL-xL at low levels compared with the other cell lines tested and were highly sensitive to 2DG and ABT-263. *MYC* expressing cells have been previously reported to be sensitive to glycolysis inhibition by 2DG, which can be attenuated by overexpression of BCL-2 [35]. In the remaining cell lines studied, ABT-263 significantly increased sensitivity to 2DG and led to a rapid reduction in viability when used in the presence of both 2DG and metformin. Furthermore, blockade of caspase activity with z-VAD-FMK inhibited cell death following treatment with ABT-263 and 2DG or the combination of all three agents. Further detailed studies are required in order to determine how ABT-263 promotes apoptosis in response to agents targeting tumour metabolism and how these agents affect the balance of pro and anti-apoptotic BCL-2 proteins in pediatric glioma cells. However, these data indicate that expression of anti-apoptotic BCL-2 family proteins in these cells is an important mechanism of resistance to metabolic therapies in pediatric glioma.

Pediatric high grade glioma is biologically distinct from adult disease and refractory to conventional multimodal therapy which is associated with significant, long term deleterious effects in survivors [1], [2]. Malignant glioma cells are frequently reported to be dependent upon aerobic glycolysis. However, recent evidence has challenged this view, demonstrating that whilst glioblastoma cells primarily metabolize glucose to lactate *in vitro*, glucose is also extensively utilised by the mitochondria *in vivo*. Similarly, glioma stem cells have been shown to be less glycolytic than differentiated glioma cells *in vitro*, having lower basal rates of lactate production, decreased uptake of glucose analogs and higher ATP levels.

Here, we show for the first time, that metformin in combination with 2DG attenuates the growth of pediatric glioma cells *in vitro*. Our data also demonstrates that combined inhibition of glycolysis and BCL-2/BCL-xL function effectively promotes cell death in response to metformin treatment in pediatric glioma cells. Moreover, these treatments are effective against glioblastoma cells featuring mutant *H3F3A* (G34V), which is present in 31% cases of pediatric glioblastoma and over 70% of cases of diffuse intrinsic pontine glioma [25], [26]. 2DG has been reported to increase sensitivity to radiotherapy in patients with glioblastoma with minimal side effects [36], [37]. 2DG and metformin have been safely and effectively combined in mice [21], [22] however, the feasibility of this drug combination with ABT-263 needs to be carefully assessed in future studies. Glioblastomas preferentially express hexokinase II and increased expression correlates with decreased survival [38]. It may therefore be possible to specifically target glycolysis in malignant gliomas using a hexokinase II inhibitor which could sensitize tumor cells to metformin and its combination with a BH3-mimetic such as ABT-263, whilst sparing normal brain cells, which depend upon hexokinase I [38]. Overall, our data indicates that targeting the BCL-2 family of anti-apoptotic proteins may increase sensitivity to therapies aimed at targeting tumor metabolism in pediatric glioma.

## Supporting Information

Figure S1 **Mutation analysis of pediatric glioma cell lines.** (A) Sequences of PCR primers used for the analysis of mutation of *TP53* and *H3F3A* in pediatric glioblastoma lines. (B) Results of mutation analysis in the cell line panel. Mutation positions are given according to the amino acid number in native protein sequence prior to any methionine cleavage. Identity of each cell line was confirmed by STR (short tandem repeat) profiling.
(TIF)

Method S1 **Mutation analysis.**
(DOCX)
