# Supplementary figures and images for: ABT-263 Enhances Sensitivity to Metformin and 2-Deoxyglucose in Pediatric Glioma by Promoting Apoptotic Cell Death
Source: PLoS One. 2013 May 17;8(5):e64051. doi: 10.1371/journal.pone.0064051 (PMC3656874; doi:10.1371/journal.pone.0064051)

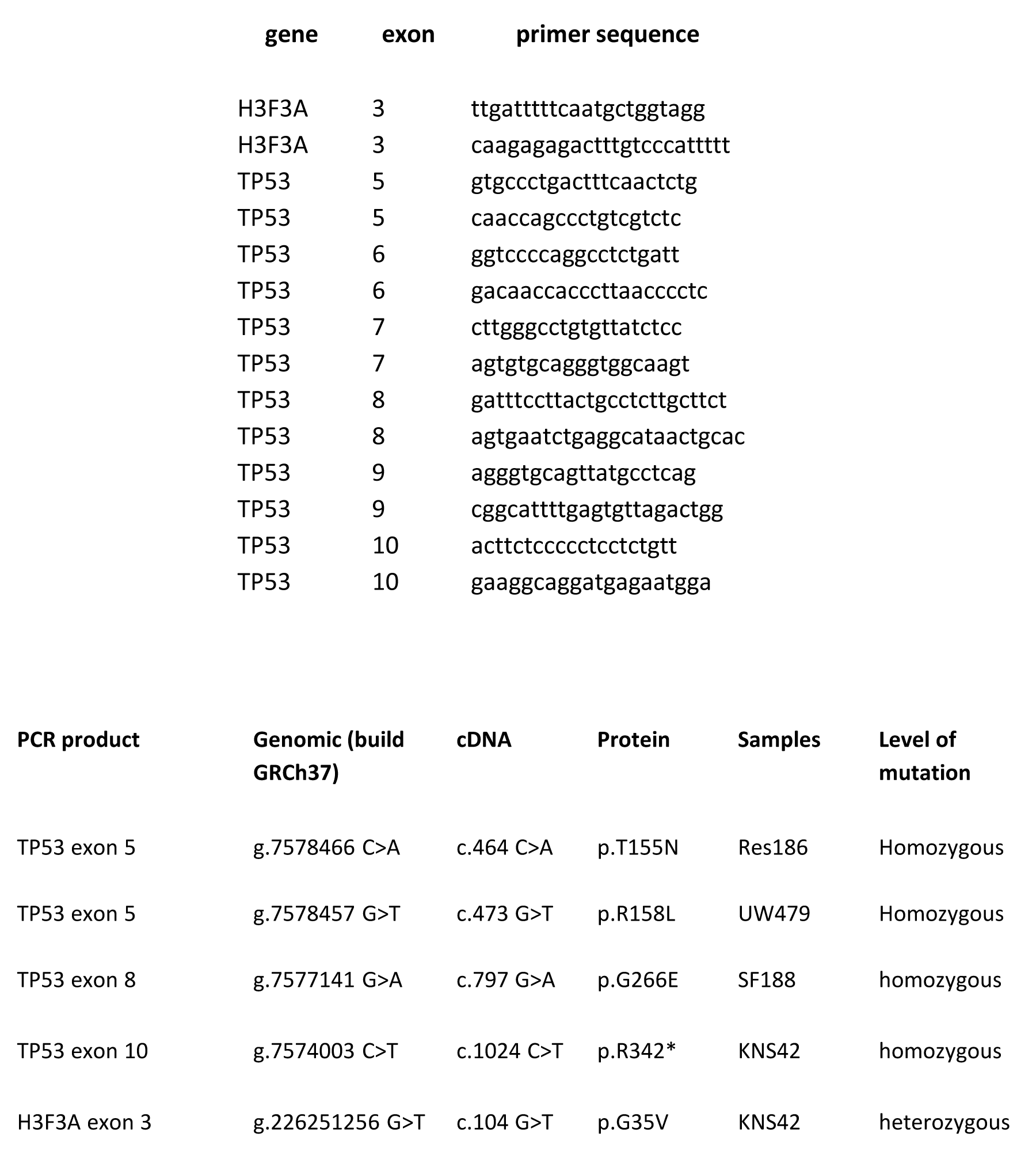

Supplement: Figure S1 — Mutation analysis of pediatric glioma cell lines. (A) Sequences of PCR primers used for the analysis of mutation of TP53 and H3F3A in pediatric glioblastoma lines. (B) Results of mutation analysis in the cell line panel. Mutation positions are given according to the amino acid number in native protein sequence prior to any methionine cleavage. Identity of each cell line was confirmed by STR (short tandem repeat) profiling. (TIF) [file pone.0064051.s001.tif]
