# Supplementary material for: ABT-263 Enhances Sensitivity to Metformin and 2-Deoxyglucose in Pediatric Glioma by Promoting Apoptotic Cell Death
Source: PLoS One. 2013 May 17;8(5):e64051. doi: 10.1371/journal.pone.0064051 (PMC3656874; doi:10.1371/journal.pone.0064051)
Supplement: Method S1 — Mutation analysis. (DOCX) [file pone.0064051.s002.docx]

**Mutation analysis**

Identity of each cell line was confirmed by STR (short tandem repeat) profiling. Mutations in *TP53*, *H3F3A*, and *IDH1* were examined by sequencing. STR profiling of cell line DNA was carried out using the PowerPlex16 HS system (Promega). Reactions were set up according to the manufacturer’s protocol; electrophoresis was carried out on a 3130xl Genetic Analyzer (Applied Biosystems) using a 36 cm capillary and POP7; and data analysis was performed using GeneMarker 1.95 (SoftGenetics). For mutation analysis, exons 5-10 of *TP53* and exon 3 of *H3F3A* were amplified by PCR using HotShot Taq master mix (http://www.clentlifescience.co.uk) in 10 µl final volume using 40 pmol of each primer and 10 ng of genomic DNA. 10% DMSO was added to all reactions except *TP53* exon 9 and *H3F3A* exon 3. Reaction conditions were 95°C for 5 minutes, 45 cycles of [95°C, 30s; 57°C 15s; 72°C, 15s]. All samples were bi-directionally sequenced using standard dye terminator chemistry. Sequencing primers were the same as the PCR primers. Data collection was performed using an Applied Biosystems 3130xl Genetic Analyzer. Data analysis was carried out by visual inspection of electropherograms and using Mutation Surveyor 3.2 (SoftGenetics).
